# Retraction: Perception exploration on robustness syndromes with pre-processing entities using machine learning algorithm

**DOI:** 10.3389/fpubh.2026.1867302

**Published:** 2026-05-06

**Authors:** Frontiers Editorial Office

The journal retracts the 16 June 2022 article cited above.

Following publication, concerns were raised regarding the validity of the data in the article. The authors failed to provide a satisfactory explanation during the investigation, which was conducted in accordance with Frontiers' policies.

This retraction was approved by the Chief Editors of Frontiers in Public Health and the Chief Executive Editor of Frontiers. The authors received a communication regarding the retraction and had a chance to respond. This communication has been recorded by the publisher.

Frontiers would like to thank the users on PubPeer for bringing the published article to our attention.

